# Prescription opioid misuse motive latent classes: outcomes from a nationally representative US sample

**DOI:** 10.1017/S2045796020000037

**Published:** 2020-01-29

**Authors:** Ty S. Schepis, A. S. De Nadai, J. A. Ford, S. E. McCabe

**Affiliations:** 1 Department of Psychology, Texas State University, San Marcos, Texas, USA; 2 Department of Sociology, University of Central Florida, Orlando, Florida, USA; 3 Center for the Study of Drugs, Alcohol, Smoking and Health, School of Nursing, University of Michigan, Ann Arbor, Michigan, USA; 4 Institute for Research on Women and Gender, University of Michigan, Ann Arbor, Michigan, USA; 5 Institute for Healthcare Policy and Innovation, University of Michigan, Ann Arbor, Michigan, USA; 6 Center for Human Growth and Development, University of Michigan, Ann Arbor, Michigan, USA

**Keywords:** Latent class analysis, misuse, motive, prescription opioid, substance use disorder

## Abstract

**Aims:**

Prescription opioid misuse (POM) contributes to a larger opioid crisis in the US and Canada, with over 17 000 US POM-related overdose deaths in 2017. Our aims were to (1) identify specific profiles of respondents based on POM motives using the US National Survey on Drug Use and Health (NSDUH) and (2) compare profile respondents on sociodemographics, substance use and mental and physical health outcomes.

**Methods:**

Analyses included 2017–18 NSDUH respondents with data on POM motives (*n* = 4810). POM was defined as prescription opioid use in a way not intended by the prescriber, including use without a prescription, in larger amounts or more frequently. Nine POM motives for the most recent episode were assessed, including ‘to relieve physical pain’ and ‘to get high’. Latent classes, based on POM motives, were estimated. Classes were compared on sociodemographics, substance use and physical and mental health outcomes.

**Results:**

Eight latent classes were identified (in order of prevalence): pain relief only, relax-pain relief, sleep-pain relief, multi-motive, high, experimenter, emotional coping and dependent/hooked. Compared to the pain relief only group, the high and multi-motive classes had higher odds of all substance use outcomes, with the dependent/hooked class having higher odds on all but one outcome. Six of the eight classes had higher odds of past-year mental health treatment and suicidal ideation than the pain relief only class.

**Conclusions:**

Screening for pain, pain conditions, problematic substance use and psychopathology are recommended in those with any POM. While those in the dependent/hooked, multi-motive and emotional coping classes are most likely to have prescription opioid use disorder (OUD), screening for OUD symptoms in all individuals with POM is also warranted.

## Introduction

Opioid misuse is a major public health issue in the US (Scholl *et al*., 2018; Vivolo-Kantor *et al*., 2018) and Canada (Abdesselam *et al*., 2018), and illicit fentanyl seizures are increasing in the European Union (European Monitoring Centre for Drugs and Drug Addiction, 2018). In the US, opioid misuse significantly contributes to increased overdose rates and decreasing life expectancy (Abdesselam *et al*., 2018; Murphy *et al*., 2018; Scholl *et al*., 2018). While the key driver of opioid overdose has shifted from prescription opioid misuse (POM) to heroin and/or illicit fentanyl use (Seth *et al*., 2018), over 17 000 US deaths were caused by POM in 2017, more than those caused by heroin (Scholl *et al*., 2018). Also, POM precedes heroin initiation in a majority of those using heroin (Compton *et al*., 2016). Furthermore, the correlates and consequences of POM are concerning and include psychopathology and significant other substance use (Martins *et al*., 2009, 2012; Fischer and Argento, 2012; Morley *et al*., 2017).

Research capturing factors associated with POM could clarify its etiology and reduce its personal and societal consequences; one such POM-related factor could be the motives, or underlying reasons for POM. Research on marijuana-related motives (Blevins *et al*., 2016) suggests that changes in motives co-occur with reductions in use, and alcohol use interventions that incorporate motives-based feedback reduce use in the short-term (Carey *et al*., 2007; Canale *et al*., 2015). Endorsement of specific POM motives may direct screening, such as screening for opioid agonist therapy in those motivated to counteract physical dependence.

Physical pain relief is the most prominent POM motive in adolescents and young adults (McCabe *et al*., 2009*a*, 2009*b*; Kelly *et al*., 2015; LeClair *et al*., 2015), with a lower prevalence of other substance use in those solely motivated by pain relief (McCabe *et al*., 2009*a*, 2009*b*, McCabe and Cranford, 2012). Only one investigation, however, has examined POM motives in US residents across age groups: Han and colleagues (2018) used the 2015 National Survey on Drug Use and Health (NSDUH) to examine the respondent's primary motive at the most recent POM episode. They found that physical pain relief was the main single motive (63.4%), with ‘to get high’ or ‘to relax’ also above 10% (11.6 and 10.9%, respectively). Any POM was associated with greater levels of substance use disorders (SUD) and suicidal ideation, but within POM motives, physical pain relief was generally associated with the lowest substance use problem prevalence (Han *et al*., 2018).

The current POM motive literature is limited by a focus on younger groups and by the use of either single motives (Han *et al*., 2018) or externally-imposed motive groupings (McCabe *et al*., 2009*b*), without any validation of such groupings via techniques such as latent class analysis (LCA) or factor analysis. LCA has been successfully applied to POM research, with Carlson *et al*. (2014) finding three classes of young adults engaged in POM, based on factors including POM frequency, motives and SUD symptoms. Their three classes had differential endorsement of POM to get high, with frequent, moderate and low endorsement by class; greater endorsement of other substance use was found in white young adults with a frequent endorsement of ‘to get high’. In addition, LCA-based examinations of POM suggest that prescription opioid SUD clusters with other SUDs and psychopathology (De Nadai *et al*., 2019) and that classes separate based on mental and physical health concerns (Cochran *et al*., 2017).

LCA may be useful in POM motive research, as individuals often have several motivations for POM, with 77% of adolescents engaged in POM endorsing more than one past-year motive (McCabe and Cranford, 2012). Single motive approaches can neglect to capture those who have many concerns driving POM engagement, whether related to pain and mental health (e.g., physical pain relief and relaxation) or avoidance of dependence symptoms. Externally-imposed groupings of motives may not correspond to real-world heterogeneity in POM motives and the associated clinical profiles. Furthermore, work is needed across the lifespan to potentially understand how POM changes through the aging process, as recent work suggests age-related changes in POM processes (Schepis *et al*., 2018).

### Aims

Our primary aim was to apply LCA to understand POM motives across the lifespan in a nationally representative US sample. After establishing a class structure, latent classes were compared on sociodemographics (e.g., sex, age group), substance use (e.g., past-month binge alcohol use), SUDs (e.g., past-year any SUD), mental health (e.g., past-year major depression) and physical health (e.g., self-reported health) outcomes. These aims were achieved through the use of the 2017–2018 NSDUH public use files.

## Methods

The NSDUH is an annual US survey of those 12 years and older, with an independent, multistage area probability design, allowing for nationally representative estimates. The NSDUH selects eligible dwelling units within US Census tracts, with a random sample of individuals from the dwelling approached to participate. To maximise data completeness and honest reporting, the NSDUH assesses sensitive topics (e.g., POM) via audio computer-assisted self-interviewing (ACASI), and it uses data imputation and consistency checks. The weighted screening response rate ranged from 75.1 to 73.3%, and the weighted interview response ranged from 67.1 to 66.6%, similar to other US nationally representative studies (Grant *et al*., 2014). Higher response rates were in 2017. More information on the NSDUH, including on psychometrics, is available elsewhere (Center for Behavioral Health Statistics and Quality [CBHSQ], 2015, 2018*a*, 2018*b*, 2019*a*, 2019*b*, 2019*c*). The NSDUH was approved by the Research Triangle International IRB (CBHSQ, 2017), and the Texas State University IRB exempted this work from further oversight.

### Participants

In the 2017–18 NSDUH public use files, 5046 respondents endorsed past-year POM; of those, 4810 (95.3%) had complete motives data and endorsed a motive other than ‘other’ (please see Measures, below). Among those with past-year POM but insufficient motives data, 157 did not respond to the motive questions and 79 only endorsed ‘other’. The sociodemographics of the analytic sample are captured in Table 1. The analytic sample was more male, whiter and less likely to graduate college, with higher proportions between 18 and 34 years, with lower household incomes and of sexual minority individuals than the entire 2017–18 NSDUH sample (*p*'s < 0.001).

**Table 1.** Sociodemographic, substance use, mental and physical health variable prevalence across participants (*n* = 4810)

	% (95% confidence interval)
Sociodemographics	
Sex	
Male	52.7 (50.6–54.8)
Female	47.3 (45.2–49.4)
Age group	
12–17	6.7 (6.1–7.3)
18–25	21.0 (19.7–22.5)
26–34	22.7 (21.2–24.2)
35–49	25.2 (23.4–27.0)
50–64	17.9 (16.0–20.0)
65 and older	6.5 (5.3–8.0)
Race/ethnicity	
White	67.5 (65.4–69.5)
African-American	10.8 (9.5–12.3)
Hispanic/Latino	15.7 (14.1–17.4)
Asian-American	2.2 (1.8–2.8)
American Indian	0.9 (0.7–1.2)
Hawaiian/Pacific	0.6 (0.3–1.0)
Multiracial	2.3 (1.8–2.9)
Income	
Under $20 000	20.5 (18.9–22.2)
$ 20 000–49 999	31.1 (28.7–33.6)
$ 50 000–74 999	16.0 (14.5–17.6)
$ 75 000 and over	32.4 (30.5–34.5)
Education	
In school	17.8 (16.7–19.0)
Less than high school	11.4 (10.3–12.6)
High school graduate	22.4 (20.8–24.1)
Some college or associate's degree	29.2 (27.3–31.2)
College graduate	19.2 (17.3–21.3)
Sexual orientation	
Heterosexual	82.8 (81.2–84.3)
Lesbian, gay or bisexual	10.4 (9.1–11.8)
Adolescents	6.7 (6.1–7.3)
Substance use	
Past-month binge alcohol use	44.2 (42.0–46.4)
Past-month daily cigarette use	25.0 (23.5–26.6)
Past-year benzodiazepine misuse	23.3 (21.6–25.0)
Past-year prescription opioid use disorder	16.4 (14.6–18.2)
Past-year any substance use disorder	41.1 (38.8–43.5)
Past-year substance use disorder treatment	9.2 (8.1–10.6)
Mental health	
Past-year major depression	21.9 (20.2–23.8)
Past-year mental health treatment	30.9 (28.8–33.2)
Past-year suicidal ideation (adults only)	16.8 (15.1–18.6)
Physical health/healthcare	
Self-reported poor/fair health	18.4 (16.8–20.3)
Past-year emergency department utilisation	39.2 (37.3–41.2)
Uninsured status	15.1 (13.6–16.8)

*Source*: 2017–18 NSUDH Surveys.

### Measures

All respondents are asked about *any opioid use*, which includes both appropriate use and misuse. To promote accurate reporting, a variety of generic and trade opioid medication names are used, and pictures of commonly used medications are provided. Those with opioid use are later asked about POM, defined as use ‘in any way a doctor did not direct…including: using it without a prescription of your own; using it in greater amounts, more often or longer than you were told to take it; using it in any other way a doctor did not direct you to use it’.

Those endorsing past-year POM were asked about motives at their last episode. These participants selected from nine potential motives, choosing as many as applied. Motives were to: relieve physical pain, relax, experiment, get high, sleep, help with emotions, alter other drug effects; ‘because I'm hooked’ and other were also included. Here, ‘other’ was not included, following precedent (Han *et al*., 2018).

Sociodemographic variables were: sex, race/ethnicity, age group, household income, educational status and sexual orientation. Sexual orientation was included due to evidence of higher POM rates among sexual minority adolescents and young adults (Dagirmanjian *et al*., 2017; Li *et al*., 2018). Substance use outcomes were: past-month POM frequency, past-month binge alcohol use, past-year marijuana use, past-year benzodiazepine misuse, past-year DSM-IV prescription opioid use disorder (OUD), past-year any DSM-IV SUD and past-year SUD treatment. Binge alcohol use is defined, per NIAAA recommendations (NIAAA, 2004), as four or five alcoholic drinks (for females and males, respectively) in one occasion. Mental health outcomes were: past-year DSM-IV major depression, past-year mental health treatment and past-year suicidality; physical health outcomes were: past-year emergency department use and self-reported poor/fair health. Finally, uninsured status was assessed.

### Analyses

Analyses occurred in Mplus 8.0 and Stata 16.0. LCA is a person-focused approach that identifies multivariate response patterns among participants. While individual variables reflect overall group averages, LCA classes reflect subgroups of participants within the set of variables. When there are multiple types of participants in a sample (e.g., when different individuals show distinct patterns of drug use motives), LCA allows for a personalised characterisation of participant responses beyond overall sample means on each item. First, we estimated latent class models in Mplus, with the eight POM motives as indicators. LCA models incorporated the NSDUH complex survey features and weighting and were estimated via robust full-information maximum likelihood. Random starts were utilised to prevent local maxima from impacting model estimation, and the best log-likelihood values were replicated for all considered models. Most likely class membership for each respondent was estimated using a modal approach, with the highest posterior predicted probability of class membership based on the model with the best fit (Collins and Lanza, 2010).

Following LCA model estimation, sociodemographic characteristic prevalence was estimated by latent class, with design-based Pearson *χ*^2^ tests (converted into *F*-values) used to evaluate differences among latent classes. Design-based logistic models estimated odds of the substance use, mental and physical health outcomes by latent class, with the pain relief class set as the reference group, given past work suggesting those motivated to engage in POM solely for pain relief have lower prevalence of substance use and other poor outcomes (McCabe *et al*., 2009*b*; Han *et al*., 2018). Finally, to investigate differences in past-month POM frequency, zero-inflated negative binomial regression analyses were performed, controlling for sociodemographics; such a model was necessitated by the high proportion of no POM in the past month and by overdispersion of the frequency data.

## Results

### Latent class model selection

Model fit indicators through an eleven-class model are provided in Table 2. We employed an iterative process to establish the ultimate number of classes, beginning with a one-class model. The one-class model fit indicators were compared to a two-class model, with each *k* model compared to the *k−*1 model. The Bayesian information criterion (BIC; Schwarz, 1978) was the indicator of model fit, with decreases in BIC values of 10 or more indicating superior model fit (Kass and Raftery, 1995). Entropy captured confidence in class separation, with entropy values above 0.80 reflecting ‘high’ class separation (Clark and Muthén, 2009). Per Masyn (2013), the final model was selected based on both model fit and interpretability. Based on model fit parameters, eight- through ten-class solutions were considered. An eight-class model was selected, given the similar model fit values and the superior parsimony and interpretability of fewer classes.

**Table 2.** Model fit criteria for latent class analysis models

Number of classes	BIC	Entropy
1	28 761.85	–
2	26 286.37	0.76
3	26 016.59	0.77
4	25 775.75	0.84
5	25 638.80	0.88
6	25 512.97	0.91
7	25 406.24	0.93
8	25 305.87	0.94
9	25 300.68	0.95
10	25 295.05	0.95

**Fig. 1.**
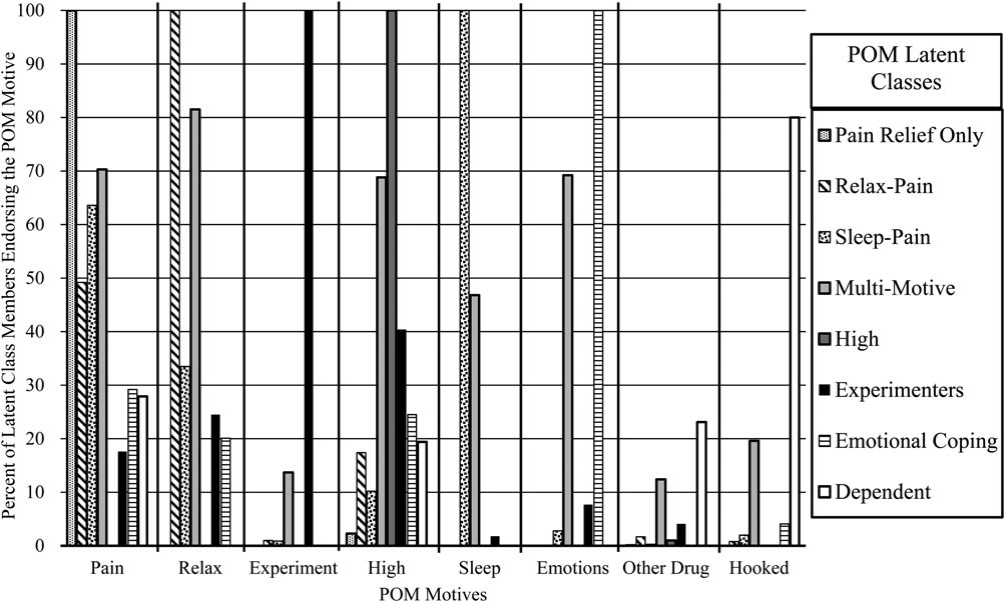
POM Motive Endorsement by Latent Class.

*Source*: 2017–18 NSUDH Surveys. POM, prescription opioid misuse.

Classes were, in decreasing order of prevalence: pain relief only (50.5%), relax-pain relief (11.9%), sleep-pain relief (11.1%), multi-motive (8.7%), high (6.4%), experimenters (4.9%), emotional coping (3.9%) and dependent/hooked (2.6%). POM motive endorsement by latent class is captured in Fig. 1. In all, 31.2% endorsed more than one motive for POM. The pain relief class was marked by 100% endorsement of physical pain relief as a motive, with 2.3% endorsement of ‘to get high’ and 0.1% of less endorsement of additional motives. In contrast, the multi-motives group was marked by the second-highest levels of endorsement for all POM motives. The relax-pain relief class was marked by 100% endorsement of relaxation and 49.2% endorsement of physical pain relief.

The sleep-pain relief class had 100% endorsement of ‘to sleep’, very high pain relief endorsement (63.6%) and elevated endorsement of ‘to relax’ (33.5%). The high class was marked by full endorsement of ‘to get high’ as a POM motive, with only 1% endorsement of another motive, to alter other drug effects. The experimenter class had 100% endorsement of ‘to experiment’ as a POM motive, with relatively high endorsement of ‘to get high’ (40.4%), and the emotional coping class was characterised by 100% endorsement of ‘to deal with emotions’ and greater than 20% endorsement of pain relief, relaxation and ‘to get high’. Finally, the dependent/hooked group had the highest endorsement of ‘because I'm hooked’, at 80%, and the highest endorsement of POM to alter other drug effects (23.1%).

### Sociodemographic characteristics of POM motive latent classes

First, sex varied by class (design-based *F*_(5.98, 299.20)_ = 9.59, *p* < 0.0001), with greater proportions of females in the sleep-pain relief, multi-motive and emotional coping classes and especially high proportions of males in the experimenter, dependent/hooked and high classes (see Tables 1 and 3). Similarly, the age group varied significantly by latent class (design-based *F*_(11.41, 570.41)_ = 6.40, *p* < 0.0001), with those 65 and older particularly concentrated in the pain relief only class and relatively lower rates in younger groups. The experimenter group was concentrated in adolescents (18.9%) and young adults (42.5%), with decreasing prevalence with age.

**Table 3.** Opioid misuse motive latent classes by sociodemographic characteristics

	Pain relief only	Relax-pain	Sleep-pain	Multi-motive	High	Experimenters	Emotional coping	Dependent/Hooked
Unweighted *N* (Weighted %)	2430 (50.5%)	572 (11.9%)	535 (11.1%)	418 (8.7%)	306 (6.4%)	235 (4.9%)	190 (3.9%)	123 (2.6%)
	% (95% CI)	% (95% CI)	% (95% CI)	% (95% CI)	% (95% CI)	% (95% CI)	% (95% CI)	% (95% CI)
Sex								
Male	51.2 (47.9–54.6)	58.1 (51.8–64.2)	43.2 (37.0–49.7)	47.5 (41.2–53.8)	72.3 (67.3–76.8)	68.0 (60.1–75.1)	34.7 (24.8–46.3)	66.6 (51.2–79.1)
Female	48.8 (45.4–52.1)	41.9 (35.8–48.2)	56.8 (50.3–63.0)	52.5 (46.2–58.8)	27.7 (23.2–32.7)	32.0 (24.9–39.9)	65.3 (53.7–75.2)	33.4 (20.9–48.8)
Age group								
12–17	5.1 (4.3–6.0)	7.2 (5.6–9.4)	4.9 (3.5–6.8)	9.0 (6.6–12.3)	10.0 (7.7–13.0)	18.9 (14.6–24.3)	8.7 (5.7–13.2)	1.3 (0.3–4.8)
18–25	15.2 (13.9–16.7)	24.3 (21.1–27.8)	18.0 (14.6–22.0)	29.5 (25.2–34.2)	39.5 (33.1–46.2)	42.5 (34.2–51.2)	21.6 (15.2–29.7)	16.8 (10.2–26.4)
26–34	21.8 (19.6–24.2)	25.7 (20.4–31.8)	20.3 (15.7–25.9)	31.1 (25.8–36.9)	20.3 (15.4–26.4)	15.4 (12.0–19.6)	21.9 (14.9–31.0)	28.3 (19.6–39.0)
35–49	27.3 (24.9–30.0)	26.5 (21.6–32.0)	25.0 (20.2–30.5)	22.3 (17.6–27.8)	23.1 (17.8–29.3)	10.7 (6.6–16.9)	20.8 (13.9–29.8)	26.9 (16.9–40.2)
50–64	20.6 (17.3–24.3)	14.8 (9.7–21.8)	24.8 (18.7–32.1)	6.6 (3.4–12.6)	4.6 (2.3–9.0)	9.4 (4.1–20.1)	25.5 (14.3–41.2)	26.7 (12.4–48.3)
65 and older	10.0 (7.7–12.8)	1.6 (0.6–4.3)	7.0 (3.1–15.2)	1.5 (0.3–6.4)	2.5 (0.3–15.8)	3.0 (0.7–12.0)	1.5 (0.4–6.2)	no cases
Race/ethnicity								
White	65.7 (62.7–68.7)	65.7 (60.5–70.4)	66.0 (59.3–72.2)	77.0 (71.1–81.9)	66.8 (60.5–72.5)	71.5 (64.9–77.2)	68.7 (57.9–77.8)	77.4 (55.7–90.3)
African–American	12.1 (10.0–14.4)	10.8 (8.2–14.0)	13.4 (9.0–19.4)	7.3 (4.8–10.9)	11.1 (7.9–15.4)	7.0 (4.0–11.9)	6.1 (3.4–10.6)	1.0 (0.1–7.0)
Hispanic/Latino	16.9 (14.9–19.1)	17.6 (14.0–21.9)	12.9 (9.7–16.9)	10.7 (7.2–15.7)	17.0 (12.2–23.1)	13.9 (9.6–19.7)	13.8 (7.4–24.4)	16.4 (5.0–42.2)
Asian–American	2.2 (1.5–3.1)	1.8 (1.0–3.4)	2.5 (1.2–5.3)	1.7 (0.7–3.7)	1.7 (0.7–4.4)	3.9 (2.3–6.5)	4.9 (1.4–15.5)	0.1 (0.02–1.1)
American Indian	1.2 (0.8–1.7)	0.7 (0.3–1.5)	0.3 (0.1–0.6)	0.3 (0.1–0.7)	1.0 (0.4–2.3)	1.6 (0.4–5.8)	0.8 (0.2–3.0)	1.1 (0.3–4.3)
Hawaiian/Pacific	0.6 (0.3–1.5)	0.3 (0.09–0.9)	0.1 (0.02–1.1)	0.8 (0.2–3.5)	no cases	0.5 (0.07–3.9)	2.6 (0.8–7.8)	no cases
Multiracial	1.4 (1.0–1.8)	3.2 (2.2–4.7)	4.8 (2.1–10.7)	2.3 (1.2–4.5)	2.4 (1.5–3.7)	1.7 (0.9–3.0)	3.1 (1.4–6.9)	4.0 (1.4–10.9)
Income								
Under $ 20 000	19.7 (17.6–22.0)	15.6 (12.6–19.2)	22.4 (16.6–29.5)	19.3 (14.5–25.1)	28.6 (22.3–36.0)	20.2 (14.9–26.9)	16.6 (11.6–23.4)	40.2 (25.8–56.6)
$ 20 000–49 999	32.4 (29.2–35.9)	32.2 (26.7–38.2)	28.7 (22.9–35.3)	33.5 (28.2–39.2)	24.6 (19.6–30.4)	26.7 (19.6–35.3)	28.3 (20.2–38.0)	29.9 (19.5–43.0)
$ 50 000–74 999	15.9 (13.5–18.5)	17.3 (13.3–22.2)	14.7 (10.9–19.4)	17.2 (13.2–22.0)	15.4 (11.2–20.9)	13.9 (8.6–21.7)	21.9 (12.5–35.3)	11.1 (5.4–21.3)
$ 75 000 and over	32.0 (29.2–35.0)	34.9 (29.2–41.1)	34.3 (27.8–41.3)	30.1 (24.3–36.5)	31.3 (24.9–38.6)	39.2 (31.7–47.3)	33.2 (23.9–44.1)	18.8 (10.2–31.9)
Education								
In school	13.9 (4.0–18.3)	18.4 (15.3–22.0)	20.6 (15.3–27.1)	22.6 (18.7–27.1)	26.4 (21.1–32.6)	34.6 (27.3–42.7)	18.6 (12.7–26.5)	8.8 (4.0–18.3)
Less than HS	11.4 (11.7–18.9)	14.9 (11.7–18.9)	9.9 (6.9–14.0)	8.8 (6.1–12.4)	11.6 (6.9–18.9)	8.3 (4.9–13.5)	12.4 (6.7–21.8)	13.5 (8.0–21.8)
HS graduate	23.1 (20.8–25.5)	19.7 (16.1–23.8)	19.1 (14.8–24.2)	18.1 (14.4–22.4)	30.7 (25.2–36.7)	14.5 (10.0–20.6)	21.6 (12.3–35.2)	46.6 (29.5–64.5)
Some college or associate's degree	31.6 (28.6–34.7)	28.2 (23.0–34.2)	26.5 (20.6–33.4)	29.5 (23.9–35.8)	18.3 (14.0–23.4)	24.8 (18.0–33.0)	34.5 (24.6–46.0)	25.6 (14.2–41.8)
College graduate	20.1 (17.3–23.2)	18.8 (14.1–24.6)	24.0 (17.7–31.5)	21.1 (16.4–26.6)	13.1 (8.6–19.3)	17.9 (11.8–26.1)	12.9 (6.4–24.0)	5.5 (1.3–20.8)
Sexual orientation								
Heterosexual	86.4 (84.2–88.2)	81.8 (78.3–84.9)	84.5 (78.6–89.1)	74.3 (69.0–79.0)	77.7 (72.1–82.5)	67.4 (60.5–73.6)	77.1 (69.1–83.5)	91.3 (82.2–96.0)
LGB	8.5 (3.3–16.1)	10.9 (8.4–14.2)	10.5 (6.6–16.4)	16.5 (12.8–21.0)	12.2 (8.3–17.5)	13.7 (9.4–19.4)	14.2 (9.0–21.6)	7.4 (3.3–16.1)
Adolescents	5.1 (4.3–6.0)	7.2 (5.6–9.4)	4.9 (3.5–6.8)	9.0 (6.6–12.3)	10.0 (7.7–13.0)	18.9 (14.6–24.3)	8.7 (5.7–13.2)	1.3 (0.3–4.8)

95% CI, 95% confidence interval; HS, high school; LGB, lesbian, gay or bisexual.

*Source*: 2017–18 NSUDH Surveys.

Race/ethnicity (design-based *F*_(14.56, 728.21)_ = 1.97, *p* = 0.016) and household income (design-based *F*_(13.41, 670.62)_ = 1.77, *p* = 0.041) significantly covaried with class membership, with only smaller deviations from the proportions of the entire sample. For educational status (design-based *F*_(15.13, 756.34)_ = 3.63, *p* < 0.0001), the class membership-educational status association was complex, though lower relative rates of high-class membership were seen in those who attended or graduated from college; high rates of experimenter class membership were found in those in school, which was consistent with the age cohort results. For sexual orientation (design-based *F*_(9.60, 479.75)_ = 7.20, *p* < 0.0001), heterosexual individuals had notably higher relative rates of pain relief only or sleep-pain class membership than lesbian, gay or bisexual (LGB) individuals, who had somewhat elevated rates of multi-motive class membership.

### Substance use outcomes by POM motive latent class

Per Table 4, those in the pain relief only class generally had significantly lower odds of current substance use and SUD diagnoses than those in other classes. Strikingly, those in the multi-motive and high classes had higher odds than those in the pain relief only class of all six substance use/SUD outcomes. Those in the multi-motive and high classes had 549 and 163% greater odds (respectively) of a past-year prescription opioid-specific SUD, 597 and 408% greater odds of any past-year SUD (respectively) than those in the pain relief class. Those in the emotional coping and dependent classes also had higher odds of the substance use/SUD outcomes than those in the pain relief class, except for past-month binge alcohol use, which was non-significant. The dependent class had the highest relative odds ratios of past-year prescription OUD [13.57, 95% confidence interval (95% CI) = 7.18–25.65], any SUD (12.1, 95% CI = 5.58–26.24) and SUD treatment (8.90, 95% CI = 4.66–17.00). While the sleep-pain relief and relax-pain relief classes had fewer significant differences, relative to the pain relief only group, these classes still had 127 (relax-pain relief) and 72% (sleep-pain relief) greater odds of any past-year SUD than those in the pain relief class (see Table 4).

**Table 4.** Substance use outcomes by opioid misuse motive latent classes

Latent class	Past-month binge alcohol use^a^	Past-month daily cigarette use	Past-year benzodiazepine misuse	Past-year prescription opioid use disorder	Past-year any SUD	Past-year SUD treatment
	AOR (95% CI)	AOR (95% CI)	AOR (95% CI)	AOR (95% CI)	AOR (95% CI)	AOR (95% CI)
Pain relief only	1.00 (reference)	1.00 (reference)	1.00 (reference)	1.00 (reference)	1.00 (reference)	1.00 (reference)
Relax-pain	1.98 (1.50–2.62)***	1.10 (0.80–1.54)	2.14 (1.55–2.96)***	1.99 (1.33–2.97)**	2.27 (1.73–2.97)***	1.14 (0.73–1.79)
Sleep-pain	1.88 (1.44–2.46)***	0.78 (0.50–1.22)	1.54 (1.05–2.26)*	1.37 (0.86–2.18)	1.72 (1.28–2.31)***	1.29 (0.61–2.73)
Multi-motive	1.55 (1.10–2.17)*	2.07 (1.48–2.89)***	4.45 (3.38–5.88)***	6.49 (4.62–9.11)***	6.97 (5.00–9.71)***	5.66 (3.76–8.53)***
High	2.30 (1.63–3.23)***	1.60 (1.16–2.20)**	4.32 (3.10–6.01)***	2.63 (1.78–3.88)***	5.08 (3.60–7.18)***	4.78 (2.88–7.94)***
Experimenters	2.96 (1.92–4.57)***	0.75 (0.44–1.25)	3.42 (2.21–5.28)***	1.12 (0.49–2.58)	3.06 (2.04–4.59)***	1.73 (0.93–3.23)
Emotional coping	1.31 (0.80–2.15)	2.99 (1.72–5.21)***	2.67 (1.83–3.91)***	4.71 (2.69–8.24)***	5.94 (3.57–9.90)***	3.93 (1.99–7.75)***
Dependent/hooked	0.75 (0.43–1.30)	3.61 (1.83–7.09)***	11.09 (5.62–21.88)***	13.57 (7.18–25.65)***	12.10 (5.58–26.24)***	8.90 (4.66–17.00)***

AOR, adjusted odds ratio; 95% CI, 95% confidence interval; SUD, substance use disorder.

*Source*: 2017–2018 NSUDH Surveys.

Logistic models control for sex, age group, race/ethnicity, household income, educational attainment and sexual orientation.

a Binge alcohol use is defined as 5 or more alcoholic drinks for men or 4 or more drinks for women in one occasion.*Denotes *p* ⩽ 0.05, **denotes *p* ⩽ 0.01 and ***denotes *p* ⩽ 0.001.

Analyses of past-month POM frequency indicated that the pain relief only group (mean = 1.52 past-month episodes) did not differ from those in the relax-pain, sleep-pain and high classes. In contrast, those who were in the dependent/hooked (*p* < 0.001), multi-motive (*p* < 0.001) or emotional coping (*p* = 0.009) classes had more POM episodes (9.00, 4.64 and 2.62, respectively), and those in the experimenter group had fewer (0.65 episodes; *p* = 0.007).

### Mental health, physical health and insurance status outcomes by POM latent class

Relative to the pain relief only group, five of eight other classes had higher odds of past-year suicidal ideation, with elevations in odds of over 200% in the emotional coping and multi-motive classes (see Table 5). Furthermore, the multi-motive, high and emotional coping classes all had elevated odds of past-year major depression and mental health treatment, *v.* those in the pain relief group. Notably, the emotional coping class had the highest relative odds of all mental health outcomes: 273% greater odds of major depression, 374% greater odds of mental health treatment and 343% greater odds of suicidal ideation.

**Table 5.** Physical health, healthcare and mental health outcomes by opioid misuse motive latent classes

Latent class	Past-year major depression	Past-year mental health treatment	Past-year suicidal ideation^a^	Self-reported poor/fair health	Past-year ED utilisation	Uninsured status
	AOR (95% CI)	AOR (95% CI)	AOR (95% CI)	AOR (95% CI)	AOR (95% CI)	AOR (95% CI)
Pain relief only	1.00 (reference)	1.00 (reference)	1.00 (reference)	1.00 (reference)	1.00 (reference)	1.00 (reference)
Relax-pain	1.26 (0.96–1.67)	1.26 (0.94–1.67)	1.64 (1.10–2.45)*	1.20 (0.82–1.78)	1.27 (0.99–1.62)	1.16 (0.87–1.55)
Sleep-pain	1.46 (0.99–2.17)	1.69 (1.19–2.41)**	1.61 (1.09–2.39)*	1.21 (0.79–1.86)	1.22 (0.90–1.66)	0.86 (0.54–1.39)
Multi-motive	3.74 (2.60–5.36)***	3.01 (2.17–4.18)***	3.22 (2.34–4.45)***	1.09 (0.75–1.59)	1.18 (0.95–1.48)	0.83 (0.62–1.13)
High	1.61 (1.05–2.45)*	1.90 (1.28–2.82)**	1.34 (0.91–1.98)	0.73 (0.41–1.29)	0.80 (0.58–1.11)	0.70 (0.47–1.04)
Experimenters	1.32 (0.93–1.87)	1.80 (1.22–2.65)**	1.66 (1.08–2.57)*	*0.58 (0.34*–1.00)*	0.78 (0.56–1.09)	*0.48 (0.29*–0.81)**
Emotional coping	3.73 (2.04–6.82)***	4.74 (2.87–7.84)***	3.43 (1.85–6.38)***	0.86 (0.43–1.73)	1.26 (0.78–2.02)	1.01 (0.51–1.97)
Dependent/hooked	1.38 (0.77–2.46)	1.47 (0.79–2.75)	1.24 (0.66–2.54)	1.58 (0.69–3.62)	0.97 (0.53–1.79)	1.00 (0.50–2.00)

AOR, adjusted odds ratio; 95% CI, 95% confidence interval; ED, emergency department.

*Source*: 2015–2016 NSUDH Surveys.Logistic models control for sex, age group, race/ethnicity, household income, educational attainment and sexual orientation.

a Suicidal ideation is assessed in adults only, and adolescents are excluded from this outcome.*Denotes *p* ⩽ 0.05, **denotes *p* ⩽ 0.01 and ***denotes *p* ⩽ 0.001.

As opposed to mental health, however, very few physical health and health insurance outcomes differed by POM motive class, per Table 5. Only the experimenter groups differed from the pain relief only group, with lower odds of self-reported poor/fair health (0.58, 95% CI = 0.34–1.00) and uninsured status in experimenters (0.48, 95% CI = 0.29–0.81; all Table 5).

## Discussion

Based on POM motives, eight latent classes were identified: pain relief only, relax-pain relief, sleep-pain relief, multi-motive, high, experimenters, emotional coping and dependent/hooked. Nearly half (50.5%) were in the pain relief only class, characterised by the near exclusive endorsement of physical pain relief as their sole POM motive. Importantly, this differed from Han *et al*. (2018), who found that 63.4% endorsed pain relief as their main motive, when allowed to select only one motive; characterising those engaged in POM by only a single, main, motive may obscure important subgroups, such as the relax-pain relief or sleep-pain relief classes found here. These classes accounted for 23.0% of respondents, reinforcing the importance of physical pain relief in POM. Nearly one-third (31.2%) of those engaged in POM had more than one motive, which was much lower than that of McCabe and Cranford (2012); this is likely due to their focus on adolescents (*v.* a general population focus here) and assessment of key motives over the past year and the NSDUH data assessing the most recent episode. Nonetheless, that study and these results suggest that approaches capturing a single motive fail to capture this complexity in a key factor leading to POM.

The remaining 26.5% of participants had elevated prevalence rates of non-pain relief motives, though the multi-motive class also had high endorsement of pain relief (70.3%). Four of these classes (i.e., emotional coping, high, dependent/hooked, multi-motive) had elevated odds of nearly all substance use/SUD and mental health outcomes. The emotional coping, dependent/hooked and multi-motive groups also had more frequent POM than those in the pain relief only class. These results correspond well with those of Carlson and colleagues (2014), who found that classes with greater endorsement of POM to get high also had greater rates of other substance use. The emotional coping class was also notable as the class with the highest odds of mental health correlates, aligning with their endorsement of POM engagement primarily to help with emotions.

While the relax-pain relief, sleep-pain relief and experimenter classes had fewer significant differences from the pain relief only class, these groups each had elevated odds of any past-year SUD and past-year suicidal ideation. Thus, it appears that the pain relief only class has the best relative profile; nonetheless, past work (Schepis and Hakes, 2011; Saha *et al*., 2016; Han *et al*., 2018) clearly indicates that those engaged in POM for any motive have higher rates of substance use and psychopathology than those not engaged in POM.

Younger respondents, multiracial and LGB individuals had lower rates of pain relief only class membership, while membership in the pain relief only class was particularly high in adults 65 years and older. The experimenter class was largely composed of adolescents and young adults, which may explain their lower rates of uninsured status, as they are likely to be covered by either insurance from parent/guardians or government sources (e.g., CHIP). Finally, the very low rates of pain relief only class membership in sexual minority respondents further highlight this vulnerable subpopulation as one in need of greater substance use prevention and intervention efforts and further study, as little work has examined sexual minority POM across the population.

### Limitations

First, the NSDUH is cross-sectional, which precludes formal causal inference. Mental health variables in particular were correlates rather than influences on class selection, despite evidence that mental health variables and POM have complicated and bidirectional relationships (Martins *et al*., 2012). Longitudinal work examining pathways leading to POM class membership and respondent changes in class membership (whether between classes or to POM abstinence) would have a great public health value. Second, self-selection bias was likely, given the refusal of some approached individuals to participate. Self-report bias was also possible, though evidence suggests that self-report substance use data are reliable and valid (O'Malley *et al*., 1983; Johnston and O'Malley, 1985). The NSDUH methodology limits self-report bias via ACASI methods, medication pictures and trade and generic medication name use (CBHSQ, 2014). Finally, given that this was a secondary data analysis, the sample and analyses are limited by the participants and measures selected or excluded for using the NSDUH. The NSDUH does not sample incarcerated or homeless individuals outside of shelters, and older adults in controlled access settings (e.g., nursing homes) are likely under sampled (Cunningham *et al*., 2015). With regard to measures, the NSDUH lacks a geographic location variable, assessments of pain, pain diagnoses and measurements of POM duration.

### Clinical implications and summary

Opioid misuse motives are complex, with a large class engaged solely for pain relief but seven other classes with combinations of motives, often also including pain relief. The specific classes suggest differential screening priorities and provide an epidemiological estimation of the relative number of individuals in each profile. Given the class distribution, the most acute need among those engaged in POM is for screening for pain and evaluation of pain management, especially among older adults. Half of those engaged in POM endorsed physical pain relief as their sole motivation for POM, and three other latent classes (i.e., multi-motive, relax-pain relief and sleep-pain relief) accounting for over 30% of the sample engaged in POM endorsed pain relief as a prominent motive.

Screening in those endorsing pain relief only as a motive may be more difficult, however, given their relatively lower rate of problematic substance use and psychopathology; thus, these individuals may be less likely to seek substance abuse or mental health treatment than members of other latent classes. As such, screening for POM in those with pain-related complaints may have utility. Attention to pain management should not preclude screening for signs of OUD, psychopathology and other substance use in those only endorsing pain relief, as these individuals have much higher rates of these correlates than those without POM (Han *et al*., 2018).

Screening for OUD, psychopathology and other problematic substance use is also needed in the other latent classes. While those in the relax-pain relief and sleep-pain relief classes have somewhat lower relative odds of other substance use and psychopathology, they are still at elevated risk above those without POM. Much like those in the pain relief only class, they may not present as often for treatment as members of other classes, or they may present strictly with mental health complaints. Attention to the wide spectrum of potential risk behaviours and other poor outcomes is needed, despite the potential focus on pain and mental health symptoms in these classes.

In contrast, individuals in the emotional coping, high, dependent and multi-motive classes may be more likely to seek mental health and/or substance use treatment, providing a screening opportunity. As with the other latent classes, screening for OUD, mental health and other substance use is recommended. When screening indicates treatment needs, behavioural health interventions to address psychopathology and/or substance use treatment, possibly including opioid agonist therapy, may be needed. For those in the experimenter class, early intervention (given their younger age) to prevent the entrenchment of POM and further engagement in other substance use is needed; the younger age of this group should not preclude robust intervention, as these individuals may progress into a more concerning latent class as they age.

Most importantly, *any* POM marks greater likelihood of other problematic substance use and psychopathology (Schepis and Hakes, 2011; Saha *et al*., 2016; Han *et al*., 2018), *v.* those without current POM. Thus, it is important to screen for the potential OUD, substance use and behavioural health needs of those in the pain relief only class, in addition to attention to pain management needs. In all, this work found eight latent classes engaged in POM, based on motives. Furthermore, the specific patterns of motives and motive overlap suggest that assessment of all POM motives may be important, as these patterns can indicate the relative substance use and behavioural health treatment needs of the respondent.

## Availability of data and materials

The NSDUH data are available at the Substance Abuse and Mental Health Data Archive (SAMHDA): https://www.datafiles.samhsa.gov/study-series/national-survey-drug-use-and-health-nsduh-nid13517
